# Comparison of pregnancy outcome after fresh embryo transfer between GnRH antagonist and GnRH agonist regimens in patients with thin endometrium

**DOI:** 10.3389/fmed.2023.1071014

**Published:** 2023-01-19

**Authors:** Depeng Zhao, Rui Xie, Xuemei Li

**Affiliations:** Department of Reproductive Medicine, Affiliated Shenzhen Maternity and Child Healthcare Hospital, Southern Medical University, Shenzhen, China

**Keywords:** pregnancy outcome, endometrial thickness, GnRH antagonist, GnRH, ovarian stimulation

## Abstract

**Objective:**

To compare the pregnancy outcome after fresh embryo transfer between GnRH antagonist and GnRH agonist regimens in patients with thin endometrium.

**Methods:**

This retrospective study included all fresh embryo transfers following GnRH agonist or GnRH antagonist protocols in patients with thin endometrium from 2016 to 2021. The thin endometrium was defined as an endometrial thickness of 7.5 mm or less on the triggering day. Multivariant regression analysis was applied to assess the association of GnRH agonist or GnRH antagonist regimen with live birth following fresh embryo transfer in patients with thin endometrium.

**Results:**

A total of 69 and 192 cases were, respectively, included in the GnRH antagonist or GnRH agonist group. The stimulation duration was significantly longer by the GnRH agonist protocol than the GnRH antagonist protocol (11.2 ± 2.1 vs. 9.1 ± 1.9 days, *P* = 0.002). The rates of clinical pregnancy or live birth were significantly lower in the GnRH antagonist group compared to the GnRH agonist group (26.1 vs. 47.9%, *P* = 0.027; 17.4 vs. 40.1%, *P* = 0.01, respectively). Multivariable regression analysis demonstrated that GnRH agonist regimen was related to higher live birth rate compared with GnRH agonist protocol [adjusted OR: 2.6, 95% confidence intervals (CI): 1.3–5.3]. No significant difference in miscarriage rate and the neonatal outcome was present between the two protocols.

**Conclusion:**

Our findings suggest that GnRH agonist protocol results in a higher rate of live birth after fresh embryo transfer than GnRH antagonist protocol in patients with thin endometrium.

## Introduction

The establishment of pregnancy depends on good quality embryos, endometrial receptivity and the synchronization of these two factors. Endometrial receptivity plays a pivotal role in embryo implantation and pregnancy. Various indicators including morphology and omics data are used to evaluate the endometrial receptivity. In daily practice, endometrial thickness functions as a simple and non-invasive sign for endometrial receptivity before embryo transfer. Thin endometrial thickness occurs in 2.4–8.5% of patients undergoing assisted reproductive technology and increases the adversity of pregnancy outcome (1, 2), though the uniform definition of thin endometrium is lacking. Thin endometrium usually refers to an endometrial thickness of less than 8 mm on the trigger day of fresh cycle or the progesterone addition day of frozen embryo thawing cycle (3–11).

Several studies reported that thin endometrium is associated with miscarriage, ectopic pregnancy, preterm birth and low birth weight (4, 12–17). Endometrium thickness on the day of human chorionic gonadotropin (hCG) trigger is well recognized as a predictor for live birth after fresh embryo transfer. Physicians usually choose not to proceed with the treatment cycle, counsel cycle cancelation, and subsequent frozen embryo transfer (FET) with patients with thin endometrium. However, the endometrial thickness may not be improved in subsequent FET cycles, leading to a longer treatment duration compared to fresh embryo transfer. In addition, little is known on factors associated with the pregnancy outcome after fresh embryo transfer in patients with thin endometrial thickness. Song et al. reported that fresh embryo transfer subsequent to GnRH agonist prolonged protocol yielded a higher live birth rate than the short-acting GnRH agonist long regimen (36.5 vs. 20.8%, respectively) (18), raising the hypothesis that controlled ovarian stimulation protocols might affect the pregnancy outcome following fresh embryo transfer in patients with thin endometrium. The two main protocols for controlled ovarian stimulation are GnRH agonist and GnRH antagonist regimens. Therefore, this study aims to compare the pregnancy outcome after fresh embryo transfer between GnRH antagonist and GnRH agonist regimens in patients with thin endometrium.

## Materials and methods

### Study design and patients

**FIGURE 1:**
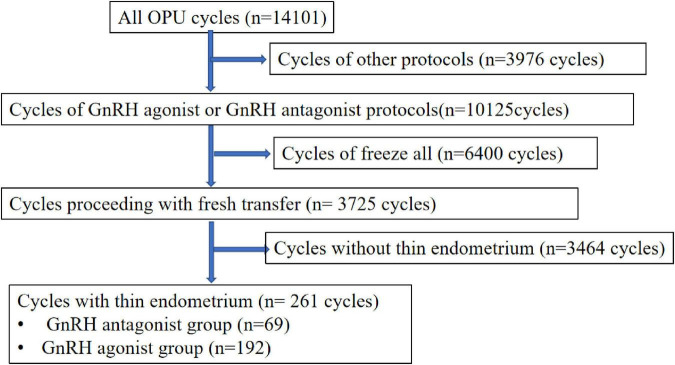
Flow chart can of the study. This figure describing flow sheet of enrolled patients.

This retrospective study performed at the department of reproductive medicine, Shenzhen Maternity and Child Healthcare Hospital from January 2016 to December 2021. All patients who underwent *in vitro* fertilization (IVF) or intracytoplasmic sperm injection (ICSI) treatment and received fresh embryo transfer were eligible for this study. The inclusion criteria of the study were as follows: first, controlled ovarian hyper-stimulation are GnRH antagonist protocol or GnRH agonist protocol; second, age between 20 and 40 years; third, the first ovarian stimulation cycle; forth, fresh embryo transfer cycles; fifth, the number of embryos transferred ≤2. The exclusion criteria included: (1) other ovarian stimulation regimens; (2) stimulation cycles for pre-implantation genetic diagnosis; (3) free-all cycles. The flow chart of patient inclusion in the study is shown in Figure 1. Patients receiving hysteroscopy surgery for intrauterine adhesion was not excluded. In this study, the exposure measure was a thin endometrium on the triggering day. Since uniform definition of thin endometrium is lacking, recognized criteria for thin endometrium ranges from 6 to 8 mm (3, 4, 7, 11). In this study, we used 7.5 mm as a cut-off for the diagnosis of thin endometrium (16). The endometrial thickness was calculated as the maximal distance from one endometrial–myometrial interface to the other one in the midsagittal plane by ultrasonography.

The Medical Ethics Committee of Shenzhen Maternity and Child Healthcare reviewed and approved this study (SFYLS [2020] 067).

### Controlled ovarian stimulation (COH) regimens

The option of COH stimulation regimen was based on anti-Müllerian hormone (AMH) test, antral follicle count (AFC), age, body weight and physician’s preference. The procedure of GnRH agonist and antagonist regimens was described as below. In GnRH agonist regimen, long-acting GnRH agonist for down-regulation of pituitary gland was injected at a dosage of 1.0–1.8 mg in the mid-luteal phase. The down-regulation of pituitary gland was assessed 14 days after administration of long-acting GnRH agonist. The criteria for successful down-regulation of pituitary gland were present as follicle diameter <8 mm, serum estradiol level <50 pg/mL, luteinizing hormone (LH) <5 IU/L and endometrium <5 mm. In case of reaching down regulation, injection of recombinant FSH (rhFSH) started with a dosage from 75 to 300 IU. In GnRH antagonist protocol, injection of rhFSH at a dosage of 75 to 300 IU was initiated on the day 2 to 4 of the menstrual cycle. The GnRH antagonist at a daily dosage of 250 mg was added when the dominant follicle was larger than 11–12 mm. In case of the diameter of two or more follicles larger than 18 mm, hCG triggering was administrated at a dose of 4,000 to 10,000 IU. Oocyte aspiration was performed at 35–38 h after hCG injection. Transfer of Cleavage-stage embryo or blastocyst proceeded on the 3rd or 5th days after fertilization. Embryo quality was evaluated according to the Istanbul consensus workshop on embryo assessment (19). In brief, the cleavage embryos on day 3 was assessed based on blastomere symmetry and fragmentation and described as good, fair or poor. Cleavage embryos scored as “good” with a blastomere count more than 7 were regarded as high-quality embryos. Blastocysts on day 5 or 6 were evaluated according to the trophectoderm and the inner cell mass (ICM), the degree of expansion of the blastocyst cavity and the status of the trophectoderm breakings out of the zona pellucida. All embryos transferred were at least good quality (Grade B). Luteal support was initiated on the day of oocyte aspiration with utility of Dydrogesterone (10 mg tid) and P suppository (Cyclogest, 400 mg bid) or 8% Crinone gel (90 mg qd). If pregnant, luteal support continued to 10 weeks of gestation.

### Follow up of infertility outcomes

In this study, hospital-registry data were used to retrieve infertility outcomes. The first serum hCG test for pregnancy confirmation was scheduled 14 days after fresh embryo transfer. If pregnant, patients usually received ultrasound examination around 30 days after fresh embryo transfer. The primary outcome measure was the live birth rate. Other infertility outcomes were also recorded, including biochemical pregnancy, clinical pregnancy, miscarriage, gestational age at birth and birth weight.

### Statistical analysis

Categorical data were expressed as frequency and percentage. Continuous variables were displayed as mean standard deviation (SD). We applied Shapiro–Wilk test to evaluate the normality of continuous variables. Student’s *t*-test or Kruskal-Wallis test was used to compare continuous variables. We applied Chi-square test or Fisher’s exact test to analyze the categorical data. Multivariable logistic regression model was built to evaluate the independent association of stimulation regimens with live birth adjusting for potential confounding factors. Adjusted odds ratios (aORs) and 95% confidence intervals (CIs) were calculated. IBM SPSS Statistics 22.0 (IBM Corporation, Armonk, NY, USA) was used to perform statistical analyses. Statistical significance was considered when a *P*-value was < 0.05.

## Results

A total of 14,101 women underwent IVF or ICS treatment at our center during the study period. After applying the exclusion criteria, 69 women who underwent a first GnRH antagonist protocol and 192 women who underwent a GnRH agonist protocol were included, as shown in the flow chart in Figure 1.

Baseline characteristics in the two groups were presented in Table 1, including maternal age, Body mass index (BMI), the rate of primary infertility, infertility duration, AMH, basal FSH, AFC, estradiol and progesterone level on the hCG trigger day (pg/ml), endometrium thickness on triggering day and infertility cause. There was no statistically significant difference in clinical characteristics between the two groups.

**TABLE 1:** Clinical baseline.

Demographics	GnRH antagonist group (*n* = 69)	GnRH agonist group (*n* = 192)	*P*-value
Female age, years	34.9 ± 5.1	33.8 ± 4.6	0.07
BMI, kg/m^2^	21.8 ± 2.7	21.8 ± 2.8	0.64
Primary infertility, *n* (%)	25 (36.2)	64 (33.3)	0.66
Duration of infertility, years	2.7 ± 2.3	3.7 ± 2.8	0.001
AMH, ng/ml	2.7 ± 2.3	3.4 ± 2.7	0.07
Basal FSH, IU/L	8.7 ± 4.7	8.3 ± 3.9	0.39
Antral follicle count	9 ± 7	11 ± 4	0.54
Endometrium thickness on triggering day, mm	6.6 ± 0.9	6.7 ± 0.7	0.86
Estradiol on triggering day, pg/mL	1983 ± 1294	2333 ± 1302	0.04
Progesterone on triggering day, ng/mL	1.02 ± 0.85	0.85 ± 0.34	0.86
**Infertility factors**			
Tubal factor, *n* (%)	31 (44.9)	72 (37.5)	0.28
Endometriosis, *n* (%)	2 (2.9)	9 (4.7)	
Polycystic ovarian syndrome, *n* (%)	4 (5.8)	8 (4.2)	
Decreased ovarian reserve, *n* (%)	5 (7.2)	6 (3.1)	
Male factor, *n* (%)	2 (2.9)	13 (6.8)	
Combined factors, *n* (%)	15 (21.7)	63 (32.8)	
Unexplained factors, *n* (%)	10 (14.5)	21 (10.9)	

### Outcomes of controlled ovarian hyper-stimulation

Table 2 shows ovarian stimulation procedures of the two protocols. The GnRH agonist protocol group had significantly higher duration of stimulation (11.2 vs. 9.1 days, *P* = 0.002), the number of oocytes retrieved (9.5 vs. 7.0, *P* = 0.001) and the Number of MII oocytes (8.5 vs. 6.2, *P* = 0.001) than the GnRH antagonist protocol group. While the GnRH agonist protocol group yielded a higher estradiol level (2333 vs. 1983 pg/mL, *P* = 0.04) on the hCG day than the GnRH antagonist protocol group. The GnRH antagonist protocol group yielded a higher total gonadotropin dose (2690 vs. 1802 pg/mL, *P* = 0.001) than the GnRH agonist protocol group. Each patient was transferred with one or two good quality embryos only and the rate of one embryo transferred was 21.7% in the GnRH antagonist protocol group was higher than the GnRH agonist protocol group (9.9%). Other results including the number of embryos available for transfer, the rate of cleavage-stage embryo transferred, and LH level on the hCG day were also similar.

**TABLE 2:** Comparison of ovary stimulation and embryo transfer between GnRH antagonist group and GnRH agonist group.

	GnRH antagonist group (*n* = 69)	GnRH agonist group (*n* = 192)	*P*-value
Days of ovarian stimulation per cycle	9.1 ± 1.9	11.2 ± 2.1	0.002
Total gonadotropin dose (IU) per cycle	2690 ± 1368	1802 ± 1583	0.001
Total number of oocytes per cycle	7.0 ± 4.3	9.5 ± 5.2	0.001
Number of MII oocytes per cycle	6.2 ± 3.9	8.5 ± 4.6	0.001
Total number of embryos available for transfer per cycle	3.6 ± 2.3	3.9 ± 2.2	0.19
Mean number of embryos transferred	1.8 ± 0.4	1.9 ± 0.3	0.012
One embryo transferred	15 (21.7%)	19 (9.9%)	
Two embryos transferred	54 (78.3%)	173 (90.1%)	
Cleavage-stage embryo transfer	66 (95.7%)	190 (99%)	0.22

### Pregnancy outcomes

The live birth rate was 17.4% (12/69) in the GnRH antagonist protocol group and 40.1% (77/192) in the GnRH agonist protocol group, *P* = 0.001 (Table 3). The clinical pregnancy rate of the latter group were also significantly higher than that of the former group (47.9% vs. 26.1%, *P* = 0.002). There were no significant differences in the miscarriage rate, gestational age at birth and birth weight between the two protocols (*P* > 0.05). In multivariant regression analysis, applying GnRH agonist protocol contributes to a significantly higher live birth after fresh embryo transfer in patients with thin endometrium (OR 2.5, 95% CI 1.23–5.15, Table 4).

**TABLE 3:** Pregnancy outcome after embryo transfer by stimulation protocols in patients with thin endometrium.

	GnRH antagonist group (*n* = 69)	GnRH agonist group (*n* = 192)	*P*-value
Positive hCG, *n* (%)	22 (31.9)	97 (50.5)	0.005
Biochemical pregnancy, *n* (%)	4 (5.8)	5 (2.6)	0.39
Clinical pregnancy, *n* (%)	18 (26.1)	92 (47.9)	0.027
Miscarriage per clinical pregnancy, *n* (%)	5 (27.8)	15 (16.3)	0.25
Live birth, *n* (%)	12 (17.4)	77 (40.1)	0.01
Gestational age at birth, weeks	38.6 ± 1.5	37.9 ± 2.2	0.35
Birth weight, grams	3166 ± 822	2930 ± 731	0.39

**TABLE 4:** Multivariate regression analysis of live birth by stimulation protocol.

	Cycle with live birth (*n* = 89)	Cycle without live birth (*n* = 172)	OR	95% CI
GnRH antagonist protocol, *n* (%)	12 (13%)	57 (33%)	Reference	Reference
GnRH agonist protocol, *n* (%)	77 (87%)	115 (67%)	2.6	1.3–5.3
Female age, years	32.8 ± 3.9	34.8 ± 5.1	0.92	0.92–0.98
Estradiol level on triggering day, pg/ml	2465 ± 1191	2127 ± 1353	1.0	1.0–1.0
Number of retrieved oocytes, *n*	10.0 ± 5.3	8.2 ± 4.9	1.0	0.9–1.1
Antral follicle count, *n*	11 ± 6	10 ± 7	1.0	0.9–1.1

OR adjusted for female age, estradiol level on triggering day, number of retrieved oocytes and antral follicle count. The selection of confounders was based on univariate analysis (Supplementary Table 1).

## Discussion

Sufficient endometrial thickness is a known key factor for embryo implantation. While how to manage patients with thin endometrium remains a challenge for physicians. It has been suggested that control ovarian stimulation protocols were associated with pregnancy outcomes. Our findings showed that compared with GnRH antagonist, the GnRH agonist protocol is associated with higher rates of positive HCG (31.9 vs. 50.5%), clinical pregnancy (26.1 vs. 47.9%) and live birth (17.4 vs. 40.1%) in patients with thin endometrium. After adjusting for potential confounders, GnRH agonist protocol independently improved the live birth rate (OR, 2.6, 95% CI 1.3–5.3).

The thin endometrium is a great challenge for infertility treatment and decreases the live birth rate after fresh embryo transfer. Although several studies were comparing the clinical outcomes of different control ovarian stimulation protocols (20, 21), few focused on patients with thin endometrium. A single-center retrospective cohort study in 2020 was conducted to compare the live birth rate and clinical pregnancy rate of 148 GnRH-a prolonged protocol and 154 the short GnRH-a long protocol in 302 patients with endometrium <8 mm who received fresh embryo transfer. The results showed that the clinical outcome of the GnRH-a prolonged protocol was better than that of the short GnRH-a long protocol in patients with endometrium <8 mm (18). In a 2021 study by Amir et al., IVF/ICSI patients undergoing fresh transplantation were divided into GnRH-a luteal long protocol (*n* = 3104) and GnRH antagonist protocol (*n* = 1527) groups (22). According to the endometrial thickness on the trigger day, they were divided into group of endometrium thickness ≤7 mm and group of endometrium thickness from 7 to 10 mm. After propensity score matching, the clinical outcomes of the GnRH antagonist protocol and the GnRH-a luteal long protocol were comparable in clinical pregnancy, live birth, and abortion rates (22). This discrepancy may be due to the application of long or short-acting agonist, the different study population (mean age of around 34 vs. 30 years) and various causes for infertility.

It is unclear why the GnRH agonist protocol yields a higher live birth rate after fresh embryo transfer than the GnRH antagonist protocol. The endometrial receptivity rather than thickness may be the key point. Several studies argue that a long-acting GnRH agonist regimen in the follicular phase may increase pregnancy outcomes by improving both endometrial receptivity and thickness (18, 23). A recent study substantiated that GnRH agonist improves endometrial receptivity by directly regulating the expression of related enzymes and cytokines (24). Further investigation unraveled that administration of higher doses of long-acting GnRH agonist enhances the expression of endometrial receptivity-related genes such as HOXA10, MEIS1, and LIF (18, 22, 25).

Our study has several limitations. First, given the retrospective nature of this study, endometrial thickness was not considered for the application of controlled ovarian stimulation protocol. Second, there was a considerable variation in the number of patients in each group, which may compromise the accuracy of statistics. Furthermore, the etiology for thin endometrium is incompletely determined and may be heterogenous in these patients. Whether the association exists of causes for thin endometrium with pregnancy outcome remains to be elucidated.

In conclusion, our findings suggest that the GnRH agonist protocol results in a higher rate of live birth after fresh embryo transfer compared to the GnRH antagonist protocol in patients with thin endometrium, indicating the GnRH agonist protocol might be more suitable in these patients.

## Data availability statement

The raw data supporting the conclusions of this article will be made available by the authors, without undue reservation.

## Ethics statement

The studies involving human participants were reviewed and approved by the Institutional Review Board of the Center for Reproductive Medicine of Shenzhen Maternity and Child Healthcare Hospital, Affiliated to Southern Medical University. Written informed consent for participation was not required for this study in accordance with the national legislation and the institutional requirements.

## Author contributions

DZ, RX, and XL contributed to the study design, data analysis, and manuscript preparation. DZ and RX handled the patient recruitment and data collection. All authors read and approved the final manuscript, made a substantial, direct, and intellectual output to the work, and approved it for publication.
