# Supplementary material for: Comparison of pregnancy outcome after fresh embryo transfer between GnRH antagonist and GnRH agonist regimens in patients with thin endometrium
Source: Front Med (Lausanne). 2023 Jan 19;10:1071014. doi: 10.3389/fmed.2023.1071014 (PMC9892192; doi:10.3389/fmed.2023.1071014)
Supplement: Supplementary file 1 [file Table_1.docx]

**Supplemental Table 1 Clinical characteristics stratified by live birth status.**

| Variates | Live birth (n=89) | Non-live birth (n=172) | P value |
| --- | --- | --- | --- |
| Female age, years | 32.8±3.9 | 34.8±5.1 | <0.001 |
| BMI, kg/m2 | 21.6±2.8 | 21.9±2.7 | 0.391 |
| Basal FSH, IU/L | 8.0±2.6 | 8.6±4.7 | 0.137 |
| Antral follicle count, n | 11±6 | 10±7 | 0.150 |
| GnRH agonist protocol, n(%) | 77 (86.5) | 115 (66.9) | 0.001 |
| Endometrium thickness on triggering day, mm | 6.8±0.6 | 6.6±0.8 | 0.47 |
| Estradiol on triggering day, pg/mL | 2465±1191 | 2127±1353 | 0.04 |
| Progesterone on triggering day, ng/mL | 0.90±0.29 | 0.88±0.61 | 0.77 |
| Number of retrieved oocytes, n | 10.0±5.3 | 8.2±4.9 | 0.03 |
